# Evaluating ECG Criteria for Diagnosing Left Ventricular Hypertrophy in Anabolic-Androgenic Steroid Users

**DOI:** 10.1016/j.jacadv.2025.102499

**Published:** 2026-01-14

**Authors:** Rang Abdullah, Astrid Bjørnebekk, Lisa E. Hauger, Ingunn R. Hullstein, Thor Edvardsen, Kristina Haugaa, Vibeke M. Almaas

**Affiliations:** a University of Oslo, Oslo, Norway; b ProCardio Center for Research Based Innovation, Department of Cardiology, Rikshospitalet, Oslo University Hospital, Oslo, Norway; c Anabolic Androgenic Steroid Research Group, Section for Clinical Addiction Research, Division of Mental Health and Addiction, Oslo University Hospital, Oslo, Norway; d The National Centre for Epilepsy, Division of Clinical Neuroscience, Full Member of European Reference Network on Rare and Complex Epilepsies EpiCARE, Oslo University Hospital, Oslo, Norway; e Norwegian Doping Control Laboratory, Department of Pharmacology, Oslo University Hospital, Oslo, Norway

**Keywords:** anabolic-androgenic steroids, ECG, left ventricular hypertrophy

## Abstract

**Background:**

Anabolic-androgenic steroid (AAS) users represent a high-risk cardiovascular population. Left ventricular (LV) hypertrophy (LVH) is a common complication, yet the utility of electrocardiographic (ECG) criteria for detecting LVH remains unexplored.

**Objectives:**

The objective of the study was to evaluate the diagnostic performance of six commonly used ECG-LVH criteria in identifying echocardiographic LVH among long-term AAS users.

**Methods:**

We included 100 male AAS users, 32 with echocardiographic LVH and 68 without. Six ECG-LVH criteria (Sokolow-Lyon, Modified Sokolow-Lyon, Cornell voltage, Cornell product, Peguero-Lo Presti, and Romhilt-Estes) were assessed for sensitivity, specificity, and predictive values, using echocardiography as the reference standard. LV systolic function was evaluated by LV ejection fraction and global longitudinal strain.

**Results:**

AAS users with echocardiographic LVH exhibited more pronounced LV systolic dysfunction than those without, as shown by a higher number of individuals with LV ejection fraction ≤40% (7 [23%] vs 4 [6%], *P* = 0.02) and more impaired LV global longitudinal strain (−14.7% ± 2.7% vs −16.5% ± 2.2%, *P* = 0.002). Among voltage-based indices, Peguero-Lo Presti provided the best balance between sensitivity (31%) and specificity (88%). The point-based Romhilt-Estes using a cutoff of 5 offered comparable performance, with a sensitivity of 34% and specificity of 85%. However, most AAS users with echocardiographic LVH were not identified by ECG, with 69% remaining undetected by Peguero-Lo-Presti and 66% by Romhilt-Estes.

**Conclusions:**

ECG was insufficient for identifying echocardiographic LVH in AAS users who represent a high-risk cardiovascular population. Routine specialist evaluations, including echocardiography, should be considered for long-term AAS users to facilitate early detection of LVH and associated dysfunctions.

Anabolic-androgenic steroids (AAS) constitute a group of compounds that includes the male sex hormone testosterone, along with its various synthetic derivatives. Although the use of AAS is illegal in most Western countries, it remains widespread among men seeking to enhance physical appearance and athletic performance.^1^ Once predominantly limited to elite athletes, AAS use has expanded in the last 5 decades to the general population, with higher prevalence seen in subpopulation such as recreational weightlifters, bodybuilders, inmates, and patients with substance use disorders.^2^ The spread of AAS use among the general population is partly caused by the online sales on the Internet, with recent systematic reviews estimating a lifetime prevalence of AAS use in Europe and the United States at around 2% to 3%.^3^

In recent years, the association between AAS use and cardiovascular toxicity has become well documented, partly attributable to the increasing number of long-term users of AAS. Our previous research has shown that long-term AAS use is associated with a cardiac phenotype characterized by myocardial hypertrophy, and impaired left (LV) and right ventricular systolic function, indicating a biventricular cardiomyopathy, with a high occurrence of severely reduced ejection fraction.^4^ The LV systolic function was observed also in former users, which may indicate permanent changes in function. Comparable findings are seen in other studies, including evidence of coronary artery disease and impaired vascular function among users of AAS.^5^, ^6^, ^7^, ^8^ Furthermore, recent large register studies have found higher incidence of cardiovascular events^9^ and an increase in mortality among AAS users.^10^^,^^11^

Although the relationship between AAS use and LV hypertrophy (LVH) is well documented, few studies have investigated the corresponding changes that may be observed on an electrocardiography (ECG). In clinical practice, the ECG remains the first-line screening test for evaluation of patients with suspected heart disease, owing to its widespread availability and low cost.^12^ Signs of LVH on an ECG are associated with increased cardiovascular morbidity and mortality, as demonstrated by the Framingham Study.^13^ Additionally, ECG signs of LVH (ECG-LVH) are correlated with both the development^14^ and detection^15^^,^^16^ of LVH and impaired LV systolic function, as confirmed by echocardiography. Although echocardiography is a reliable method for diagnosing LVH, it requires operator expertise and is less accessible than ECG. Despite the importance of detecting LVH in AAS users, the utility of ECG-determined LVH for identifying high-risk patients within this population remains unaddressed. This study aims to evaluate the diagnostic performance of various ECG criteria in relation to echocardiography as reference standard in long-term users of AAS.

## Methods

### Study design and population

This cross-sectional study is part of a longitudinal study investigating the relationship between high-dose AAS use and brain health, carried out at the Anabolic Androgenic Steroid Research Group at Oslo University Hospital, Norway (https://www.ous-research.no/anabolic-steroids/). The participants, which include men who engage in intense strength training, were recruited from this project between May 2017 and October 2019. The sample and recruitment methods are described in detail in previous brain health publications.^17^^,^^18^ As compensation, participants received a 500 NOK (approximately $60) gift card. The sample comprised current and former (defined as at least 1 year since cessation of use) users of AAS who were enrolled in a previously published cardiovascular study.^4^ The echocardiographic data used in this study were previously collected and published as part of the aforementioned study. Participants were allocated into 2 groups for further analysis: those with echocardiographic “LVH” and those without (“no LVH”). The study was conducted in accordance with the Declaration of Helsinki and received ethical approval from the Regional Committee for Medical and Health Research Ethics in South-Eastern Norway (2013/601). Participants received an informational brochure with a complete description of the study before participation, and written informed consent was obtained from all participants.

### Screening, instruments, clinical interview, and examination

Relevant health and background data were gathered through self-report questionnaires and clinical interviews by 2 investigators (A.B. and L.E.H.). Participants were asked about their alcohol intake, smoking habits, and illicit drug use. For illicit substances, a series of questions were posed to determine if participants had ever used specific drugs (yes or no). AAS users were questioned regarding their history with AAS use as well as details on whether and when they had discontinued the use. Blood pressure was measured in an outpatient clinic after a minimum of 15 minutes of seated rest using an automatic sphygmomanometer, with a standard bladder cuff with most patients, and larger cuffs for patients with bigger arm circumferences. If patients were hypertensive, measurements were repeated and reported as the average of the last 2 readings.

### Doping analysis

Urine samples were collected and analyzed for external use of androgens using gas and liquid chromatography coupled to mass spectrometry at the World Anti-Doping Agency–accredited Norwegian Doping Laboratory at Oslo University Hospital.^19^ The criteria used to determine external androgen use were: 1) urine samples positive for synthetic anabolic androgenic steroids compounds; and 2) a testosterone to epitestosterone ratio >15 equivalent to the previous work.^18^, ^19^, ^20^, ^21^ In addition, the urine samples were analyzed for narcotics using Norwegian Doping Laboratory at Oslo University Hospital’s routine method.

### ECG

**Figure 1:**
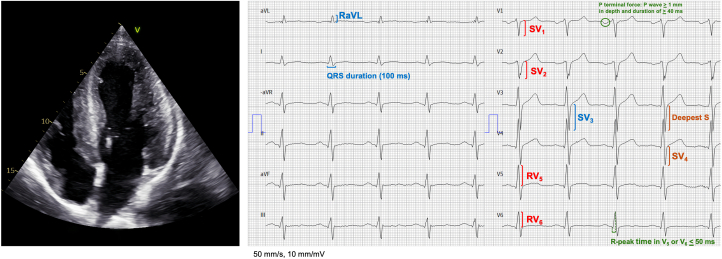
Sample Echocardiography and Electrocardiogram Echocardiographic 4-chamber view of an AAS user with moderate hypertrophy (left ventricular mass index = 138 g/m^2^). Note that none of the ECG-LVH criteria are met: Sokolow-Lyon (SV_1_ + RV_5_/V_6_; 8 + 12 = 20 mm), modified Sokolow-Lyon (SV_1_/V_2_ + RV_5_/V_6_; 10 + 12 = 22 mm), Cornell voltage (SV_3_ + RaVL; 14 + 4 = 18 mm), Cornell product (Cornell voltage x QRS duration; 18 × 100 = 1800 mm × ms), Peguero-Lo Presti (Deepest S any lead + SV_4_; 10 + 15 = 25 mm), and Romhilt-Estes score (Any limb R/S wave ≥20 mm or SV_1_/V_2_ ≥30 mm or RV_5_/V_6_ ≥30mm, ST-T changes typical of LVH, P terminal force in V_1_ ≥1 mm depth and ≥40 ms width, left axis deviation, R-peak time V_5_/V_6_ ≥50 ms; 0 + 0 + 3 + 0 + 1 + 0 = 4).

We obtained 12-lead ECGs for analysis, calibrated to 50 mm/s and 1 mV/10 mm. The ECGs were analyzed observer-blinded to clinical data and echocardiographic results, by 1 investigator (R.A.). We applied the 2 most commonly used ECG criteria for LVH in clinical practice, as found in the LIFE (Losartan Intervention for Endpoint Reduction) study,^22^ in addition to 3 other commonly reported ECG criteria for LVH (Figure 1). The ECG criteria analyzed are as follows.

1. Sokolow-Lyon index, SV_1_ + (RV_5_ or RV_6_) ≥35 mm
2. Modified Sokolow-Lyon index, SV_1_ or SV_2_ + RV_5_ or RV_6_ ≥35 mm
3. Cornell voltage, SV_3_ + RaVL >28 mm
4. Cornell product, Cornell voltage (mm) x QRS duration (ms) >2,440 mm x ms
5. Peguero-Lo Presti index,^23^ deepest S in any lead + SV_4_ ≥28 mm
6. Romhilt-Estes point score system^24^

### Transthoracic echocardiography

Echocardiography was performed using Vivid E95 (GE Vingmed Ultrasound), on the same day the ECGs were taken. All echocardiographic measurements were obtained by 1 investigator (V.M.A.) and analyzed offline, blinded to AAS status, by another investigator (R.A.) using the software EchoPAC v203 (GE). Two-dimensional echocardiographic measurements were performed according to the current standards.^25^ LV ejection fraction (LVEF) was calculated by modified the Simpson biplane method (LVEF). LV systolic function was further assessed by LV global longitudinal strain (LVGLS) using speckle-tracking in the 3 apical views and defined as the average of peak longitudinal strains from a model with 16 LV segments. If 2 or more segments in any given view were deemed inappropriate for strain assessments, LVGLS was excluded. Maximal wall thickness was assessed from parasternal short-axis view from all LV segments from base to the apex of the LV. LV mass was estimated from parasternal views using the formula provided by Devereux et al.^26^ LV mass was further indexed to body surface area (BSA) using the Dubois formula.^27^ LVH was defined as LV mass/BSA >117 g/m^2^ (LV mass indexed to BSA [LVMI]), in accordance with recent European Association of Preventive Cardiology/European Association of Cardiovascular Imaging recommendations on evaluation of the athlete’s heart.^28^ LVH was classified by additional LV geometric measurements, using a two-tiered classification system based on LVMI and relative wall thickness, in line with American Society of Echocardiography and the European Association of Cardiovascular Imaging guidelines.^29^ Sensitivity analyses were performed by repeating the main analyses using LV mass indexed to height^2.7^. A cutoff of 48 g/m^2.7^, adapted for athletic cohorts in line with European Society of Cardiology guidelines^30^ was applied to account for ECG effects attributable to body weight.

### Statistical analyses

Continuous data are presented as mean ± SD. Proportions are reported as count and percentage. Normality was assessed with Shapiro-Wilks test and visual inspection of histograms and Q-Q plots. Between-group analyses comparing the 2 independent groups were performed using Student’s *t*-test or Mann-Whitney *U* test depending on the data distribution. Proportions between groups were compared using Pearson chi-square test or Fisher exact test, depending on expected cell counts. We used ECG criteria as continuous variables to obtain area under the curve to estimate the predicted performance of the tested criteria. We calculated sensitivity, specificity, positive, and negative predictive value for each ECG criterion. The McNemar test was used to assess for lack of agreement comparing ECG criteria with echocardiographic LVH. All statistical analyses were performed using SPSS (version 29.0; IBM corp. 2024).

### Reproducibility

Reproducibility of ECG-LVH criteria and echocardiographic LV mass measurements was assessed in 10 randomly selected participants, with observers blinded to all clinical data, as well as to ECG and echocardiography results. The intraclass correlation coefficient (ICC) was calculated using a two-way mixed effects model with absolute agreement.

## Results

### Clinical characteristics

We included 100 AAS users, 32 users with echocardiographic LVH (LVH) and 68 without (no LVH) (Central Illustration). The LVH group had on average a higher height, higher body mass index (BMI) (*P* < 0.001), and higher diastolic blood pressure (*P* = 0.04), but no further differences were observed. A testosterone to epitestosterone ratio >15 was observed in 19/31 participants with LVH and 25/62 participants without LVH (*P* = 0.06) (Table 1). History of cocaine and amphetamine use was common in both groups, but mostly limited to previous exposure.

**Central Illustration:**
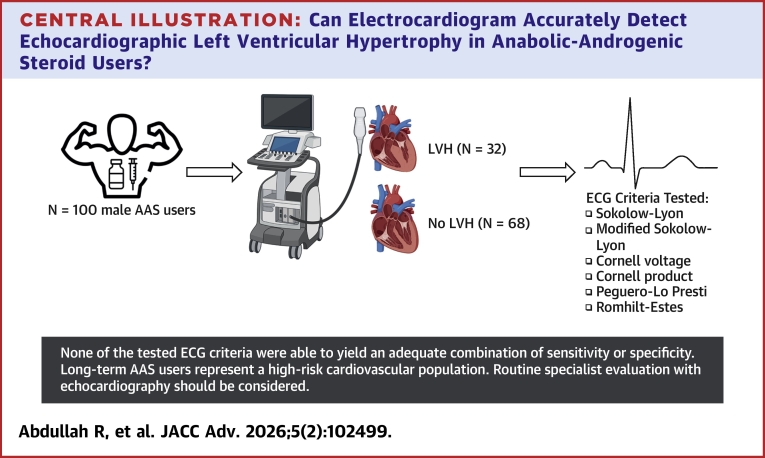
Can Electrocardiogram Accurately Detect Echocardiographic Left Ventricular Hypertrophy in Anabolic-Androgenic Steroid Users? Created in BioRender. Abdullah R (2025), https://BioRender.com/zoerbmc. AAS = anabolic-androgenic steroids; ECG = electrocardiogram; LVH = left ventricular hypertrophy.

**Table 1:** Clinical Characteristics of 32 AAS Users With Echocardiographic Left Ventricular Hypertrophy (LV Mass/BSA >117 g/m^2^) and 68 AAS Users Without

	LVH (n = 32)	No LVH (n = 68)	*P* Value
General characteristics			
Age (years)	37 ± 11	39 ± 10	0.34
Height (cm)	179 ± 8	183 ± 7	0.008
Weight (kg)	103 ± 18	97 ± 13	0.06
BMI (kg/m^2^)	32 ± 5	29 ± 3	<0.001
BSA (m^2^)	2.2 ± 0.2	2.2 ± 0.2	0.64
Height^2.7^ (m^2,7^)	4.8 ± 0.6	5.1 ± 0.5	0.009
Systolic BP (mm Hg)	146 ± 17	140 ± 17	0.10
Diastolic BP (mm Hg)	80 ± 9	76 ± 10	0.04
Drugs and alcohol			
History of smoking, n (%)	7 (22)	25 (37)	0.16
Alcohol units (week)	4.3 ± 3.9	3.4 ± 3.0	0.58
History of amphetamine use, n (%)	14 (44)	33 (49)	0.66
Current amphetamine use, n (%)	1 (7)	3 (9)	0.78
History of cocaine use, n (%)	15 (47)	33 (49)	0.88
Current cocaine use, n (%)	0 (0)	1 (3)	1.0
Anabolic-androgenic steroid use			
Former users, n (%)	6 (9)	24 (35)	0.08
Accumulated duration of AAS use (years)	13 ± 7	10 ± 7	0.16
Weekly AAS dose used (mg)	1,187 ± 839	956 ± 543	0.17
Estimated lifetime AAS dose (g)	747 ± 791	462 ± 462	0.08
T/E ratio >15, n (%)	19 (61)	25 (40)	0.06

Values are mean ± SD, unless otherwise indicated.

AAS = anabolic-androgenic steroids; BMI = body mass index; BP = blood pressure; BSA = body surface area; LV = left ventricular; LVH = left ventricular hypertrophy; T/E = testosterone to epitestosterone.

### ECG

All participants had sinus rhythm, and none had atrioventricular or complete bundle branch block. PQ, QRS, and QTc lengths did not differ between the cohorts. AAS users with echocardiographic LVH compared with no LVH showed a higher heart rate (*P* = 0.05) and higher scores of both Cornell criteria, Peguero-Lo Presti and Romhilt-Estes. Although a higher amount of AAS users with echocardiographic LVH had positive ECG-LVH using Peguero-Lo-Presti and Romhilt-Estes criteria, no such differences were noted for the other criteria (Table 2). Five AAS users had left axis deviation, and three AAS users had repolarization abnormalities, characterized by ST-segment depression and T-wave inversion or otherwise commonly referred to as “LV strain pattern.”

**Table 2:** Resting ECG of 32 AAS Users With Echocardiographic Left Ventricular Hypertrophy (LV Mass/BSA >117 g/m^2^) and 68 AAS Users Without

	LVH (n = 32)	No LVH (n = 68)	*P* Value
Heart rate (beats/min)	75 ± 14	69 ± 13	0.05
PQ duration (ms)	160 ± 22	159 ± 20	0.94
QRS duration (ms)	100 ± 8	97 ± 10	0.10
QT_c_ duration (ms)	403 ± 28	403 ± 23	0.95
LV strain pattern, n (%)	1 (3)	2 (3)	0.96
Left axis deviation, n (%)	2 (6)	3 (4)	0.69
Sokolow-Lyon (mm)	27 ± 8	27 ± 7	0.98
Modified Sokolow-Lyon (mm)	31 ± 9	29 ± 8	0.42
Cornell voltage (mm)	15 ± 6	12 ± 6	0.006
Cornell product (ms x mm)	1,540 ± 600	1,161 ± 646	0.005
Peguero-Lo Presti (mm)	24 ± 9	19 ± 8	0.004
Romhilt-Estes	3.9 ± 2.5	2.5 ± 2.0	0.007
ECG-LVH, n (%)			
Sokolow-Lyon ≥35 mm	4 (13)	8 (11)	0.94
Modified Sokolow-Lyon ≥35 mm	11 (34)	18 (26)	0.42
Cornell voltage >28 mm	1 (3)	1 (1)	0.58
Cornell product >2,440 ms × mm	3 (9)	2 (3)	0.17
Peguero-Lo Presti ≥28 mm	10 (31)	8 (11)	0.02
Romhilt-Estes ≥4	21 (66)	24 (35)	0.004
Romhilt-Estes ≥5	11 (34)	10 (15)	0.02

Values are mean ± SD, unless otherwise indicated.

ECG-LVH = left ventricular hypertrophy diagnosed by ECG; other abbreviations as in Table 1.

### Echocardiography

The echocardiographic findings of the entire population are detailed in a previous report.^4^ Comparisons of AAS users with LVH vs no LVH showed a higher prevalence of LVEF ≤40 among the former, alongside a significant difference in systolic function by LVGLS (Table 3).

**Table 3:** Baseline Echocardiographic Parameters of 32 AAS Users With Left Ventricular Hypertrophy (LV Mass/BSA >117 g/m^2^) and 68 AAS Users Without

	LVH (n = 32)	No LVH (n = 68)	*P* Value
Left ventricular dimensions			
LVMI (g/m^2^)	138 ± 13	91 ± 15	<0.001
Normal geometry, n (%)		53 (78)	
Concentric remodeling, n (%)		15 (22)	
Concentric hypertrophy, n (%)	23 (72)		
Eccentric hypertrophy, n (%)	9 (28)		
MWT (mm)	16.8 ± 3.1	12.6 ± 2.6	<0.001
IVSD (mm)	13.8 ± 2.5	10.7 ± 1.8	<0.001
PWD (mm)	12.1 ± 2.2	9.8 ± 0.2	<0.001
EDD (mm)	5.5 ± 0.7	5.2 ± 0.5	0.01
ESD (mm)	4.1 ± 0.9	3.9 ± 0.5	0.25
Left ventricular systolic function			
LVGLS (%)^a^	−14.7 ± 2.7	−16.5 ± 2.2	0.002
LVEF (%)^b^	48 ± 9	50 ± 6	0.14
EF ≤40, n (%)	7 (23)	4 (6)	0.02
EF 41-49, n (%)	9 (29)	25 (37)	0.39
EF ≥50, n (%)	15 (48)	37 (56)	0.48
EDV (mL)	163 ± 42	146 ± 31	0.06
ESV (mL)	86 ± 35	73 ± 17	0.06

Values are mean ± SD, unless otherwise indicated.

EDD = end-diastolic diameter; EDV = end-diastolic volume; EF = ejection fraction; ESD = end-systolic diameter; ESV = end-systolic volume; IVSD = interventricular septum thickness in end-diastole; LVEF = left ventricular ejection fraction; LVGLS = left ventricular global longitudinal strain; LVMI = left ventricular mass indexed to body surface area; MWT = maximal wall thickness; PWD = posterior wall thickness in end-diastole; other abbreviations as in Table 1.

a 25 AAS users with LVH and 49 AAS users without LVH were available for analysis.

b 31 AAS users with LVH and 66 AAS users without LVH were available for analysis.

### Assessment of ECG criteria

**Figure 2:**
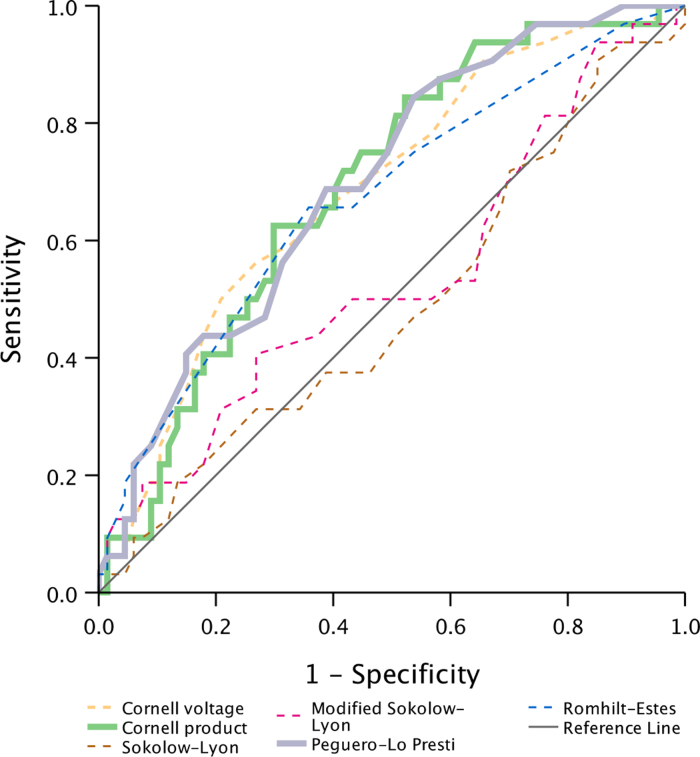
Area Under the Curve of 100 Anabolic-Androgenic Steroid Users Area under the curve of the tested ECG criteria, analyzed as continuous variables, representing the predictive performance of left ventricular hypertrophy among AAS users.

Receiver operating curve analyses demonstrated that the Cornell voltage, Cornell product, Peguero-Lo Presti, and Romhilt-Estes criteria showed moderate discriminatory ability, with nonsignificant differences among them, based on the CIs (Table 4, Figure 2). Among the voltage-based criteria, the Peguero-Lo Presti index provided the best balance of sensitivity (31%; 95% CI: 17-48) and specificity (88%; 95% CI: 79-94), although the McNemar test indicated significant discordance with echocardiography (*P* = 0.02) (Table 5). The Romhilt-Estes point score system with a threshold of ≥4 points yielded the highest sensitivity (66%; 95% CI: 48%-80%), with moderate specificity (65%; 95% CI: 53%-75%), although with borderline disagreement (*P* = 0.04) with echocardiography. Increasing the cutoff to ≥5 points improved specificity (85%; 95% CI: 76%-92%), at the expense of lower sensitivity (34%; 95% CI: 20%-52%), and disagreement with echocardiography was no longer significant (*P* = 0.07). Sensitivity analyses using LV mass indexed to height^2.7^ with a cutoff of 48 g/m^2.7^ for LVH showed no difference in ECG-LVH criteria performance compared with indexing to BSA (Supplemental Tables 1 to 4).

**Table 4:** Area Under the Curve for the ECG Criteria Predictive Performance of Left Ventricular Hypertrophy (LV Mass/BSA >117 g/m^2^) Among 100 AAS Users

	Area Under the Curve (95% CI)	*P* Value
Sokolow-Lyon	0.49 (0.37–0.61)	0.86
Modified Sokolow-Lyon	0.54 (0.42–0.67)	0.49
Cornell voltage	0.69 (0.58–0.80)	0.002
Cornell product	0.70 (0.59–0.80)	0.002
Peguero-Lo Presti	0.70 (0.60–0.81)	0.001
Romhilt-Estes	0.67 (0.55–0.79)	0.006

Area under the curve analyses of ECG criteria, analyzed as continuous variables against echocardiographic LVH.

Abbreviations as in Table 1.

**Table 5:** Performance of ECG Criteria Against Left Ventricular Mass Index, Evaluated by Sensitivity, Specificity, Predictive Values, and McNemar Test Among 100 AAS Users

	Sensitivity (95% CI)	Specificity (95% CI)	Positive Predictive Value (95% CI)	Negative Predictive Value (95% CI)	McNemar Test^a^
Sokolow-Lyon	13 (4-27)	88 (79-94)	33 (11-61)	68 (58-77)	<0.001
Modified Sokolow-Lyon	34 (20-52)	74 (62-83)	38 (22-56)	70 (59-80)	0.75
Cornell voltage	3 (0-13)	99 (94-100)	50 (4-96)	68 (59-77)	<0.001
Cornell product	9 (2-23)	97 (91-100)	60 (20-92)	70 (60-78)	<0.001
Peguero-Lo Presti	31 (17-48)	88 (79-94)	56 (33-77)	73 (63-82)	0.02
Romhilt-Estes					
≥4	66 (48-80)	65 (53-75)	47 (33-61)	80 (68-89)	0.04
≥5	34 (20-52)	85 (76-92)	52 (32-73)	73 (63-82)	0.07

Thresholds applied for ECG-LVH criteria are based on cut-off values reported in the original literature, as noted in Methods.

Abbreviations as in Table 1.

a A *P* value of <0.05 indicates a lack of agreement.

### Reproducibility

The interobserver and intraobserver ICC are presented in Supplemental Table 4. For echocardiographic LV mass, intraobserver ICC was 0.88 (95% CI: 0.55-0.97), and interobserver reproducibility was 0.91 (95% CI: 0.68-0.98). For ECG-LVH criteria, intraobserver and interobserver ICC exceeded 0.90 for all criteria.

## Discussion

This study aimed to evaluate the efficacy of using ECG as a screening tool for identifying high-risk patients with echocardiographically confirmed LVH among AAS users. A the cardiovascular toxicity of AAS is well documented, our study uniquely explores this association in detail by applying a wide range of various ECG criteria. Typically, LVH is diagnosed in a clinical setting by echocardiography with measures of LVMI. The corresponding electrical changes commonly observed on an ECG include increased cardiac voltages, left axis deviation, and ST-segment depression and T-wave inversions, making the ECG a viable and low-cost first-line screening tool. The need for effective screening is underscored by our findings. First, echocardiographic LVH was frequently observed in AAS users. In our cohort, 32% of AAS users, including former users, displayed LVH, reiterating the importance of screening in this population. Second, we discovered that LV systolic function was commonly severely impaired in those with echocardiographic LVH, indicating that AAS users with LVH are high-risk cardiovascular patients. Third, our results suggest that a large proportion of high-risk AAS users with echocardiographic LVH will remain undiagnosed if relying solely on ECG as a screening tool, as none of the trialed ECG criteria were able to yield adequate specificity and sensitivity.

### Performance of ECG criteria and clinical implications

Hypertension is common among AAS users, as shown in our cohort and several others.^7^^,^^31^^,^^32^ Although a higher afterload contributes to LV remodeling, our previous work has shown that AAS use remained the strongest determinant of LV mass after adjustment for blood pressure in multivariate analysis.^4^ Among the voltage-based indices in our cohort of AAS users, the Peguero-Lo Presti criterion demonstrated the highest diagnostic performance with a moderate area under the curve and the best balance between sensitivity and specificity. The point-based Romhilt-Estes system with a cutoff of 5 points provided comparable diagnostic performance, although its use in clinical practice is limited by its relative complexity, and reduced user-friendliness compared with simple voltage criteria. Irrespective of the specific ECG criteria applied, our findings indicate that ECG alone is a poor screening tool for detecting LVH in AAS users. This aligns with findings from a systematic review by Pewsner et al.^33^ in hypertensive patients and a recent study by Diepen et al.^34^ in elite athletes, both of which evaluated several of the same ECG criteria used in our study. This is somewhat unexpected, as AAS users are known to exhibit increased skeletal muscle fiber size on biopsy,^35^ suggesting that similar hypertrophic adaptations may occur in cardiac myocytes. Given that AAS users typically do not show myocardial fibrosis on cardiac magnetic resonance imaging^6^—as opposed to many hypertensive patients—one might expect more pronounced electrical signals and thus higher ECG voltages. The absence of such a pattern may indicate that AAS-induced hypertrophy does not translate into increased surface voltages in a predictable manner or that existing ECG criteria lack sensitivity for detecting this specific phenotype. To explore potential confounding by body composition, we conducted sensitivity analyses indexing LV mass to height^2.7^, given the known inverse relationship between BMI and QRS voltages.^36^ This adjustment did not alter the main conclusion. However, the higher BMI observed in AAS users with echocardiographic LVH compared with those without may still partly explain the poor performance of ECG-LVH criteria in this cohort. Conversely, reduced LVEF has been associated with lower ECG voltages in some studies,^37^ which may partly explain why some AAS users with echocardiographic LVH did not meet the voltage thresholds of the ECG criteria. Despite the ECG’s limit as a screening tool for echocardiographic LVH, it has been repeatedly documented that ECG-LVH is an independent risk factor for mortality, underscoring the ECG’s value in risk stratification of cardiovascular patients. Notably, a recent large-scale study has highlighted substantially elevated cardiovascular and all-cause mortality in professional male bodybuilders, reinforcing the importance of early detection and longitudinal risk assessment in this population.^38^ In addition, LVH by ECG or echocardiography has been identified as a risk factor for cardiac morbidity and mortality, independent of other risk factors including hypertension and LV mass, as documented in the Framingham Heart Study.^39^, ^40^, ^41^ LVH detected by echocardiography also detects total and cardiovascular mortality and cardiovascular events in young adults.^42^^,^^43^ The Sokolow-Lyon and Cornell criteria remain the most commonly used ECG-LVH criteria, and the 2024 European Society of Cardiology guidelines specifically recommend them as part of routine assessment of hypertensive patients.^44^ In hypertensive patients, regression of echocardiographic LVH through antihypertensive treatment has been associated with a lower likelihood of cardiovascular morbidity and mortality, irrespective of ECG changes.^45^ A similar risk-lowering effect could likely also occur in AAS users, although with the discontinuation of use. Rasmussen et al.^6^ documented significantly lower LVMI among former AAS users compared with current users, whereas in the study by Smit et al.,^46^ AAS users documented a complete recovery in LVH and LV systolic when AAS were discontinued for a median time of 8 months.

Although ECGs may be performed for various clinical reasons in AAS users, they should not specifically be relied on to identify high-risk individuals or to rule out LVH. Long-term AAS use has been associated with a wide range of cardiovascular complications, including dyslipidemia, hypertension, coronary atherosclerosis, and vascular impairment.^4^, ^5^, ^6^, ^7^ In addition, our previous work has shown that increased myocardial hypertrophy and biventricular systolic dysfunction in AAS users occur independently of factors such as athletic training, hypertension, or advanced age.^4^ For these reasons, long-term AAS users—irrespective of other cardiovascular risk factors, may benefit from specialist evaluation, including echocardiographic examination.

### Study Limitations

The limitations of this study include potential selection bias. Although participants were recruited from the community, the study population predominantly comprised White Scandinavians, which may limit the applicability of our findings to more diverse populations of AAS users. In addition, this study included only men, as they represent the majority of AAS users. As a result, it remains uncertain whether the findings are applicable to women.

LVH was assessed using echocardiography, which, although less accurate than cardiac magnetic resonance imaging,^47^^,^^48^ is the most commonly used method in clinical practice and has demonstrated good reproducibility for diagnosing LVH.^49^ However, a significant subset of AAS users in our study demonstrated ECG findings suggestive of LVH, despite negative echocardiographic results. This discrepancy highlights the potential limitation of echocardiography in detecting subtle or early structural changes. Recent studies have shown that CMR offers superior precision for identifying LVH, particularly in cases where echocardiography may produce false negative findings.^50^ It is therefore plausible that many participants classified as ECG-positive but echocardiography-negative in our study may have undetected hypertrophy that could be revealed through CMR. Although pre-existing LVH cannot be excluded, the association between AAS use and LVH has been repeatedly demonstrated. In addition, the changes that correspond with echocardiographic LVH on an ECG, are not solely dependent on the myocardium; they are also influenced by factors such as individual body composition differences, electrode placement, myocardial fibrosis, lung conditions, and other variables previously explained in detail by Bacharova et al.^47^ In AAS users, who typically also have a muscular physique, these factors can significantly affect the recorded signal on an ECG. Day-to-day changes in ECG voltages within the same patient have also been described.^51^

## Conclusions

Our findings suggest that a large proportion of AAS users with echocardiographic LVH will likely remain undiagnosed if ECG is used as a screening tool. Considering the significant cardiovascular toxicity of long-term AAS use, routine specialist evaluation with echocardiography should be considered for all AAS users, irrespective of other risk factors, to facilitate early identification and management of LVH and associated dysfunctions.

## Funding support and author disclosures

This research was funded by the 10.13039/501100006095Helse Sør-Øst RHF (grant nos. 2016049, 2017025, 2018075, and 2020088 [to Dr Bjørnebekk]) and 10.13039/501100005416The Research Council of Norway (#309762) Precision Health Center for optimized cardiac care (ProCardio). Dr Almas has received financial support from Novartis. Dr Haugaa has received financial support from Bristol Meyer Squibb, Boehringer Ingelheim Norway, Agiana Pharmaceuticals, Novartis, and Solid Bioscience. All other authors have reported that they have no relationships relevant to the contents of this paper to disclose.

Perspectives **COMPETENCY IN MEDICAL KNOWLEDGE:** This study shows that standard ECG criteria are not reliable for detecting LVH in AAS users when compared with echocardiography. Clinicians should be aware that ECG may not be a valid screening tool for LVH in this specific population.

**COMPETENCY IN PATIENT CARE:** In patients with a long history of AAS use, a normal ECG should not be considered sufficient to rule out LVH. If there is clinical concern, echocardiography should be pursued to guide diagnosis and management.

**TRANSLATIONAL OUTLOOK 1:** Further studies are needed to establish diagnostic thresholds or criteria that account for the specific cardiovascular effects of AAS use. These criteria may improve accuracy in identifying LVH through noninvasive means.

**TRANSLATIONAL OUTLOOK 2:** Exploration of population-specific ECG algorithms, potentially integrating artificial intelligence or data-driven pattern recognition, may improve the diagnostic utility of ECG in AAS users and similar nonstandard cohorts.
