# Supplementary material for: Evaluating ECG Criteria for Diagnosing Left Ventricular Hypertrophy in Anabolic-Androgenic Steroid Users
Source: JACC Adv. 2026 Jan 14;5(2):102499. doi: 10.1016/j.jacadv.2025.102499 (PMC12830151; doi:10.1016/j.jacadv.2025.102499)
Supplement: Supplemental Tables 1-4 [file mmc1.docx]

**Supplemental Table 1. Resting ECG and Echocardiography of 31 AAS users with Echocardiographic Left Ventricular Hypertrophy (LVM/m^2.7^ > 48 g/m^2.7^) and 69 AAS Users Without.**

|  | LVH (n = 31) | No LVH (n = 69) | P |
| --- | --- | --- | --- |
| **Echocardiography** | | | |
| LVM (g/m^2^) | 304 ± 42 | 201 ± 39 | <0.001 |
| LVM/m^2.7^ | 64 ± 8 | 39 ± 7 | <0.001 |
| LVM/BSA > 117 (n, (%)) | 29 (94) | 3 (4) | <0.001 |
| LVEF (%)^a^ | 47 ± 8 | 50 ± 6 | 0.04 |
| LVGLS (%)^b^ | -14.6 ± 2.9 | -16.5 ± 2.0 | 0.001 |
| **ECG** | | | |
| Heart rate (beats/min) | 74 ± 13 | 69 ± 13 | 0.09 |
| PQ duration (ms) | 163 ± 22 | 158 ± 20 | 0.28 |
| QRS duration (ms) | 101 ± 9 | 97 ± 10 | 0.05 |
| QT_c_ duration (ms) | 401 ± 25 | 404 ± 24 | 0.46 |
| LV strain pattern (n, (%)) | 1 (3) | 2 (3) | 0.93 |
| Left axis deviation (n, (%)) | 3 (10) | 2 (3) | 0.69 |
| Sokolow-Lyon (mm) | 27 ± 8 | 27 ± 7 | 0.15 |
| Modified Sokolow-Lyon (mm) | 31 ± 9 | 30 ± 8 | 0.35 |
| Cornell voltage (mm) | 16 ± 7 | 12 ± 5 | 0.002 |
| Cornell product (ms x mm) | 1595 ± 760 | 1142 ± 549 | 0.001 |
| Peguero-Lo Presti (mm) | 25 ± 10 | 19 ± 7 | <0.001 |
| Romhilt-Estes | 4.1 ± 2.7 | 2.4 ± 1.8 | <0.001 |
| **ECG-LVH (n, (%))** | | | |
| Sokolow-Lyon > 35 mm | 4 (13) | 8 (12) | 0.87 |
| Modified Sokolow-Lyon > 35 mm | 11 (35) | 18 (26) | 0.34 |
| Cornell voltage > 28 mm | 2 (6) | 0 (0) | 0.09 |
| Cornell product > 2440 ms x mm | 4 (13) | 1 (1) | 0.03 |
| Peguero-Lo Presti > 28 mm | 10 (32) | 8 (12) | 0.01 |
| Romhilt-Estes > 4 | 20 (65) | 25 (36) | 0.009 |
| Romhilt-Estes > 5 | 11 (35) | 10 (15) | 0.02 |

Data are presented as mean ± SD and n (%) when appropriate. LVH, left ventricular hypertrophy; LVM, left ventricular mass; LVM/BSA, left ventricular mass indexed to body surface area; LVEF; left ventricular ejection fraction, LVGLS, left ventricular global longitudinal strain. ^a^30 AAS users with echocardiographic LVH and 67 AAS users without LVH were available for analysis. ^b^24 AAS users with echocardiographic LVH and 50 AAS users without LVH were available for analysis.

**Supplemental Table 2. Area Under the Curve for the ECG Criteria Predictive Performance of Left Ventricular Hypertrophy (LVM/m^2.7^ > 48) Among 100 AAS Users.**

|  | Area under the curve (95% CI) | P |
| --- | --- | --- |
| Sokolow-Lyon | 0.52 (0.39; 0.65) | 0.78 |
| Modified Sokolow-Lyon | 0.55 (0.42; 0.68) | 0.45 |
| Cornell voltage | 0.68 (0.57; 0.80) | 0.002 |
| Cornell product | 0.69 (0.58; 0.80) | 0.001 |
| Peguero-Lo Presti | 0.70 (0.59; 0.80) | <0.001 |
| Romhilt-Estes | 0.68 (0.57; 0.80) | 0.002 |

Area under the curve analyses of ECG criteria, analyzed as continuous variables against echocardiographic LVH (LVM/height^2.7^ > 48).

**Supplemental Table 3.** **Performance of ECG Criteria Against Left Ventricular Mass Indexed to Height^2.7^, Evaluated by Sensitivity, Specificity, Predictive Values, and McNemar Test Among 100 AAS Users.**

|  | Sensitivity (95% CI) | Specificity (95% CI) | Positive predictive value (95% CI) | Negative predictive value (95% CI) | McNemar Test* |
| --- | --- | --- | --- | --- | --- |
| Sokolow-Lyon | 13 (4; 28) | 88 (79; 94) | 33 (12; 61) | 69 (59; 78) | 0.002 |
| Modified Sokolow-Lyon | 36 (20; 53) | 74 (63; 83) | 38 (22; 56) | 72 (61; 81) | 0.87 |
| Cornell voltage | 7 (2; 20) | 100 (95; 100) | 100 (34; 100) | 70 (61; 79) | <0.001 |
| Cornell product | 13 (4; 28) | 99 (94; 100) | 80 (37; 99) | 72 (62; 80) | <0.001 |
| Peguero-Lo Presti | 32 (18; 50) | 88 (80; 95) | 56 (33; 77) | 74 (64; 83) | 0.02 |
| Romhilt-Estes | | | | |  |
| > 4 | 65 (47; 80) | 64 (52; 75) | 44 (31; 59) | 80 (68; 89) | 0.03 |
| > 5 | 36 (20; 53) | 86 (76; 93) | 52 (32; 73) | 75 (64; 83) | 0.10 |

*A p value of < 0.05 indicates a lack of agreement.

**Supplemental Table 4. Intra- and inter-observer reproducibility**

|  | Intra-observer ICC (95% CI) | Inter-observer ICC (95% CI) |
| --- | --- | --- |
| LVM | 0.88 (0.55; 0.97) | 0.91 (0.68; 0.98) |
| **ECG-LVH Criteria** | | |
| Sokolow-Lyon | 0.98 (0.94; 1.0) | 0.98 (0.90; 0.99) |
| Modified Sokolow-Lyon | 0.99 (0.97; 1.0) | 0.98 (0.94; 1.0) |
| Cornell voltage | 0.97 (0.90; 0.99) | 1.0 (0.99; 1.0) |
| Cornell product | 0.98 (0.91; 1.0) | 1.0 (0.99; 1.0) |
| Peguero-Lo Presti | 0.99 (0.94; 1.0) | 0.98 (0.94; 1.0) |
| Romhilt-Estes | 0.98 (0.91; 0.99) | 0.92 (0.71; 0.98) |

ICC, intraclass coefficient; LVM, left ventricular mass.
